# Factors Associated with Post-COVID Cardiac Conditions and Potential Prognostic Factors: A Systematic Review

**DOI:** 10.3390/life15030388

**Published:** 2025-02-28

**Authors:** Lidian Franci Batalha Santa Maria, Josicélia Estrela Tuy Batista, Virginia Kagure Wachira, Wenderval Borges Carvalho Junior, Alexandre Anderson de Sousa Munhoz Soares, Isis Polianna Silva Ferreira de Carvalho, Henry Maia Peixoto

**Affiliations:** 1 Center of Tropical Medicine, Faculty of Medicine, Campus Universitário Darcy Ribeiro, University of Brasilia, Brasília 70910-900, Brazil; 2 Department of Strategic Actions for Epidemiology and Surveillance in Health and Environment, Secretariat of Health and Environmental Surveilance, Ministry of Health, Brasília 70719-040, Brazil; 3 University Hospital of Brasília, University of Brasília—EBSERH, Brasília 70840-901, Brazil; 4 Institute of Advanced Health Education and Research Aramari APO, Brasília 70712-903, Brazil; 5 Department of Science and Technology, Secretariat of Science, Technology, Innovation and Health Complex, Ministry of Health, Brasília 70719-040, Brazil

**Keywords:** cardiovascular system, heart diseases, COVID-19, post-acute COVID-19 syndrome, risk factors, prognosis

## Abstract

Cardiac conditions are a significant category of post-COVID conditions. The objective of this study was to synthesise the evidence on the factors associated with the development of post-COVID cardiac conditions, the frequency of clinical outcomes in affected patients, and the potential prognostic factors. A systematic review was conducted using the databases EBSCOhost, MEDLINE via PubMed, BVS, and Embase, covering studies from 2019 to December 2023. A total of 8343 articles were identified, and seven met the eligibility criteria for data extraction. The protective effect of vaccination stood out among the associated factors, showing a reduced risk of developing post-COVID cardiac conditions. Conversely, COVID-19 reinfections were associated with an increased risk of cardiovascular outcomes. Regarding the main outcomes in these patients, most recovered, although some cases persisted beyond 200 days of follow-up. The study included in the analysis of prognostic factors reported that the four children who did not recover by the end of the study were between two and five years old and had gastrointestinal symptoms during the illness. The COVID-19 vaccination regimen reduces the risk of developing post-COVID cardiac conditions. Public health policies promoting immunisation should be encouraged to prevent SARS-CoV-2 infections and reinfections.

## 1. Introduction

COVID-19, caused by SARS-CoV-2, is a multisystemic infection with both immediate and long-term effects, some of which may be subtle or consistent [1]. Its spread and severity led the World Health Organisation (WHO) to declare the disease a pandemic on 11 March 2020 [2].

The clinical course of COVID-19 typically improves after the acute phase; however, some patients experience persistent, new, or recurring symptoms following this phase [3]. In Brazil, the Ministry of Health, in line with the WHO, adopted the term “post-COVID conditions” to define these new clinical manifestations [3].

The incidence of persistent symptoms in individuals affected by COVID-19 is estimated by the WHO to be between 10% and 20% [4]. A meta-analysis found at least one persistent symptom in 45% of COVID-19 survivors, regardless of hospitalisation status [5]. But some studies report higher frequencies, especially those evaluating hospitalised patients [6].

Long-term cardiac and vascular damage has been described after infection in individuals with no prior history of cardiovascular disease [1,7], regardless of the severity of the condition or the need for hospitalisation [7]. A broad spectrum of cardiac conditions is among the post-COVID conditions, with myocarditis, heart failure [8], pericarditis, and atrial fibrillation [7] being particularly notable. Approximately 25% of individuals with post-COVID conditions may experience some form of cardiac damage, such as mild systolic dysfunction or myocarditis [8].

Screening for cardiac damage during the convalescent phase is essential to assess the burden and mitigate the impact of cardiac conditions resulting from SARS-CoV-2 infection [9]. This knowledge is crucial for guiding the development of treatment plans and preventive measures to reduce negative outcomes after the acute phase of the disease [9].

Based on existing scientific knowledge that COVID-19 patients may experience cardiac damage beyond the acute phase of the disease, regardless of pre-existing cardiovascular conditions [7], this systematic review sought to answer three research questions that could shed light on the natural history of post-COVID cardiac conditions. The objective of this study was to synthesise the evidence on the factors associated with the development of post-COVID cardiac conditions, the frequency of clinical outcomes in affected patients, and the potential prognostic factors.

## 2. Materials and Methods

A systematic review was conducted to answer three research questions: What factors are associated with the development of post-COVID cardiac conditions? What is the incidence of the primary outcomes among patients with post-COVID cardiac conditions? What are the potential prognostic factors for patients with this condition?

The three research questions were formulated according to the PECOS strategy (population, exposure, comparison, outcome, and study design), as detailed in Supplementary Table S1.

The case definition adopted was as follows: “post-COVID cardiac condition”—individuals with no prior history of heart problems who developed a cardiac condition more than four weeks after the initial COVID-19 diagnosis, or who exhibited the condition before four weeks but continued to experience it after this period.

This study was elaborated in accordance with the guidelines of the Preferred Reporting Items for Systematic Reviews and Meta-Analyses (PRISMA) [10]. The protocol for this review was registered in the International Prospective Register of Systematic Reviews (PROSPERO) on 31 May 2022, under registration number CRD42022336460 [11].

### 2.1. Search Strategy

This research consisted of two searches in the following databases: EBSCOhost Research Databases (EBSCO), MEDLINE via PubMed, Virtual Health Library (BVS), and Embase, along with searches in grey literature (Google Scholar, ProQuest, and World Wide Science) and references of the selected publications. The database queries were carried out on 1 July 2022 and updated on 16 December 2023.

The search strings included the terms “COVID-19”, “cardiac sequelae”, “post-acute COVID-19 syndrome”, “cohort studies”, “case-control studies”, “prognostic studies”, and synonyms using MeSH terms, adapted for each database (Supplementary Table S2).

A balance between sensitivity and specificity in the retrieval of publications was sought through the combination of precise terminology, broader keywords, and controlled vocabulary. The search strategies were tested and approved by the authors prior to initiating the review process.

### 2.2. Eligibility Criteria and Post-COVID Cardiac Condition

Articles that did not exclude individuals with a history of heart disease were included, as long as they allowed the analysis, either individually or by subgroups, of individuals without a history of heart conditions. Only articles in English, Spanish, French, and Portuguese, published from 2019 to the search date, were included.

Regarding the study design, only analytical observational studies (cohort and case-control) that investigated the factors associated with post-COVID cardiac conditions (question 1) and the prognostic factors of patients affected by these conditions (question 3) were included. In describing the main outcomes of these patients (question 2), the inclusion of descriptive cohorts in the selection processes was admitted (Supplementary Table S1).

Articles were excluded if they did not define post-COVID cardiac conditions and/or lacked laboratory confirmation of COVID-19, modelling studies, publications such as preprints without Ethics Committee approval, animal studies, or studies on other non-cardiac conditions. After reaching a consensus with the cardiology experts on the team, studies that explored test results without a defined clinical cardiac diagnosis were also excluded.

Studies on patients with multisystem inflammatory syndrome associated with COVID-19 in children (MIS-C) were included as long as the cardiac condition was assessed at least 28 days after the COVID-19 diagnosis.

### 2.3. Data Extraction and Methodological Assessment

The following data were extracted into a standardised spreadsheet, previously validated by the authors: publication year, country, type of publication, funding, conflicts of interest, study design, study population, number of participants, eligibility criteria, criteria for confirming COVID-19 and the cardiac condition, evaluated factors, assessed outcomes, frequency and association measures, among others.

The Newcastle–Ottawa Scale (NOS) [12] was used to assess the methodological quality of the studies included in questions 1 and 2, while the QUIPS Tool [13] was, used to evaluate the risk of bias in the prognostic studies (question 3). Both tools were used considering the specificities of each research question and adapted for this purpose, either using the star ratings (NOS) or classified as having low, moderate, or high risk of methodological quality [13].

The screening, selection, data extraction, and methodological quality assessment of the articles were performed in pairs and independently. Conflicts were resolved through consensus or, when necessary, with the participation of a third reviewer. For each of these steps, a calibration session was held to test the tools used and ensure the consistency of the review quality among the reviewers.

### 2.4. Analysis of Results

The results were described through a narrative synthesis of the findings, structured around the type of outcomes found and according to each research question of the review [14]. The outcomes were measured in frequency terms—absolute numbers and incidence rates, with or without the presentation of a 95% confidence interval (CI 95%)—or in effect measures, such as relative risk (RR), attributable risk (AR), odds ratio (OR), or hazard ratio (HR), along with their respective CI 95% and *p*-values. The significance level of 5% (*p* ≤ 0.05) was adopted in order to determine statistical significance in all analyses.

Descriptive variables of the study (population and exposure/comparison groups) were also collected. Absolute frequency (n) and relative frequency (%) were presented for categorical variables, while the mean with the standard deviation or median with the interquartile range (IQR) were provided for continuous quantitative variables.

It was not possible to conduct a meta-analysis or subgroup analysis due to clinical heterogeneity [15] across the studies in terms of outcomes, exposures, and populations, as well as the small number of included articles.

## 3. Results

### 3.1. Study Selection and Characteristics

The initial search yielded 4312 scientific publications from 1 December 2019 to 1 July 2022. An update identified 5277 publications from 1 July 2022 to 16 December 2023. The articles were initially exported to Mendeley Desktop (version 1.19.4), where duplicates were removed.

The references were then exported to the Rayyan platform [16], where additional duplicates were removed, and the screening process was conducted. A total of 444 duplicates were removed from the first search, and 802 from the second search.

**Figure 1:**
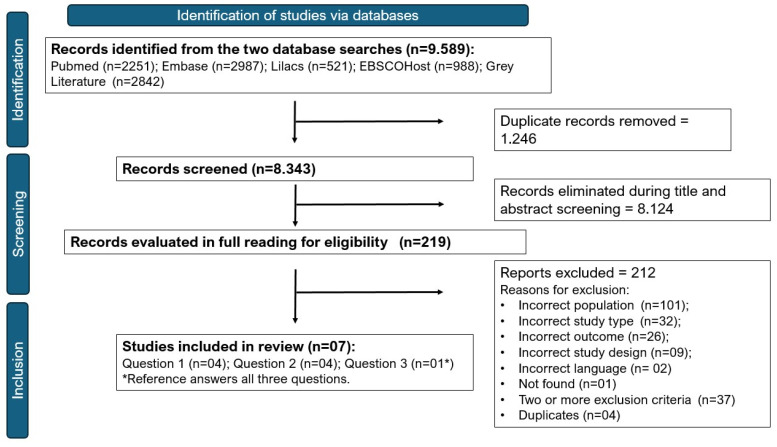
Flowchart of the evidence selection process by review stage on factors associated with post-COVID-19 cardiac condition and prognosis.

In total, 8343 references were included for title and abstract screening, following the eligibility criteria of the research. When the abstract did not clearly state the criteria, the articles were moved to the full-text review stage. In this stage, 219 publications were read. Figure 1 details the number of publications retrieved from each database, those included at each stage, and the reasons for exclusions.

After screening, seven original articles were included in the data extraction stage, all published in English. Regarding the origin of the selected articles, three are from the United States [17,18,19], one is from Hong Kong [20], one is from India [21], one is from Israel [22], and one is from the United Kingdom [23].

All manuscripts were considered cohort studies in terms of design. One study, initially classified as a case-control, was later reclassified as a cohort study since it involved follow-up of the population after outcome identification [19]. The follow-up period varied from 6 to 28 months.

The results were analysed separately for each research question. Four studies addressed the first question [17,18,20,21], and four addressed the second [19,21,22,23]. One cohort from India covered all three questions and was the only study included for the third question [21]. However, for some of the studies, only parts of the results were considered, as their objectives were broader, a pattern observed across all questions.

### 3.2. Post-COVID Cardiac Condition

All studies met the case definition adopted by this review; despite this, only three of the seven included studies characterised post-COVID conditions [17,20,22]. The most comprehensive definition described postacute sequelae of COVID-19 as new, persistent, or recurrent symptoms occurring four weeks or more after SARS-CoV-2 infection [17]. The study conducted by Shechter et al. (2022) used a 60-day period, or more, after the patients’ formal recovery (negative test) to assess outcomes [22].

Cardiac outcomes were assessed in various ways. Medical records or charts were used in a generalised manner in three studies [17,18,20]. The others relied on specific test results, such as electrocardiograms and cardiac magnetic resonance imaging (CMR) [19,21,22,23].

The type of outcome also varied across the studies. Some authors focused on grouped diagnoses of cardiac or cardiovascular diseases [17,18], while others specified the primary incident outcomes [19,20,21,22,23]. However, only cardiac diagnoses were considered in this review.

### 3.3. Factors Associated with the Occurrence of Post-COVID Cardiac Conditions (Question 1)

For the first question, 2,437,470 patients represented the total population across the four studies. This ranged from the general population (18 years or older) to military personnel and children diagnosed with MIS-C. The number of post-COVID assessments conducted during follow-up periods varied from one to seven (Table 1).

COVID-19 vaccination was assessed as an exposure in three of the four studies included [17,18,20], with two indicating a protective effect of the vaccine on the outcomes analysed [17,20]. One study found no association between the factors and the cardiac conditions studied [21].

In their study conducted in Hong Kong, Wan et al.(2023) observed that two vaccine doses protect against the occurrence of coronary artery disease, while one dose reduces the risk of heart failure (RR: 0.70; 95% CI: 0.60–0.82 and RR: 0.64; 95% CI: 0.52–0.78, respectively), regardless of age, sex, Charlson comorbidity index (CCI), disease severity, or vaccine type. For heart failure, protection was even greater with three vaccine doses (RR: 0.34; 95% CI: 0.27–0.43) (Supplementary Table S3) [20]. In addition to the number of doses, the authors compared the BNT162b2 and CoronaVac vaccines [20].

Zisis et al. (2022) conducted a study in the United States and found a reduction in the risk of heart disease in vaccinated individuals compared to non-vaccinated individuals, 28 and 90 days after COVID-19 diagnosis (RR_(28 days)_: 0.49; CI 95%: 0.43–0.57 and RR_(90 days)_: 0.35; CI 95%: 0.29–0.44) (Supplementary Table S3) [17]. This study did not declare the type of vaccine or vaccination regimen [17].

Bowe et al. (2022) simultaneously studied the effect of reinfection along with vaccination. Vaccination was assessed based on the number of doses, without specifying the type of vaccine [18]. Soldiers who were reinfected showed a higher risk of cardiovascular diseases, regardless of vaccination status, with the risk gradually decreasing over time, from 30 to 180 days after reinfection (RR_(30 to 60 days)_: 2.19; CI 95%: 1.99–2.41 and RR_(150 to 180 days)_: 1.59; CI 95%: 1.44–1.76) (Supplementary Table S3) [18].

### 3.4. Main Outcomes of Patients with Post-COVID Cardiac Conditions (Question 2)

To address the second research question, at least two measurements were required in the postacute period to assess outcomes in patients with post-COVID cardiac conditions. The studied population consisted of 202 patients with a new cardiac abnormality. Patient follow-up ranged from two to seven measurements, varying from approximately 28 to 271 days (Table 2).

Only one study observed full recovery of the patients followed, with a median of three months [22]. Another study observed active myocarditis for more than seven months after diagnosis, with 80% recovery of diagnoses by the end of follow-up, which lasted up to nine months [19]. Shah et al. (2023) noted the persistence of coronary artery aneurysm in three children, one of whom had it for more than 200 days (Table 2) [21].

### 3.5. Potential Prognostic Factors for Patients with Post-COVID Cardiac Conditions (Question 3) and Treatment Strategy

The Indian cohort by Shah et al. (2023) was the only one that met the inclusion criteria for the third question of this review [21]. The study followed 85 children with MIS-C who presented cardiac abnormalities. Forty-seven children who recovered were compared with four who did not recover. The children were aged two to five years and exhibited gastrointestinal symptoms during the illness (both with *p*-value = 0.059) [21].

One child in the non-recovery group had a persistent moderate aneurysm of the right coronary artery 182 days after the disease onset. In addition to age and symptoms, the comparison included sex, intensive care admission, shock, and the need for inotropes; however, no significant differences were observed [21].

There was a 40% loss of follow-up (34 children who were discharged clinically within three months, with undefined cardiac outcomes). A comparison between children who completed the follow-up and those who dropped out revealed differences in the presence of respiratory symptoms, which were more common among the non-adherent group (*p*-value = 0.0001). Conversely, a higher number of adherent children showed coronary artery dilation (*p*-value = 0.01) [21].

This was also the only study to describe the treatment used, which consisted of intravenous immunoglobulins (IVIGs), corticosteroids, and aspirin, along with supportive care [21]: all 144 children received oral aspirin, 78 received immunoglobulin administration at a dose of 2 g/kg (single dose), and 84 were administered steroids (dose based on phenotype) [21].

A pulse of methylprednisolone at 10 mg/kg/day for three days, followed by low-dose steroids, was administered for those who went into shock. For the children who did not present shock characteristics, low-dose steroids were administered at 2 mg/kg/day, with a gradual reduction, and low molecular weight heparin (two patients), anakinra (four patients), and tocilizumab (two children) were also given [21].

### 3.6. Assessment of the Methodological Quality of the Studies

The articles from questions 1 and 2 were evaluated using NOS for cohort studies (Table 3).

In analysing factors associated with post-COVID cardiac conditions, only one article met all the tool’s criteria [20]. The lowest score in the other articles was for item S1 (representativeness of the cohort exposed to the risk factor). Only one study did not meet the item regarding the control of confounding factors, as it did not describe whether any method was used to control them [21].

The remaining studies used at least one statistical technique to control for variables (Supplementary Table S3). Techniques such as weighting and matching were used to identify and remove possible confounders [17,18,20]. Logistic regression, and sensitivity analyses sought to detect sources of possible spurious biases [18,20], and Wan et al. (2023) also performed paired and blinded statistical analyses to avoid information bias [20].

Of the articles that analysed the primary outcomes of these patients (included in question 2), none met all the tool requirements, with the cohort’s representativeness in criterion S1 as the only item not awarded a star in any of the studies. All studies scored on the items related to outcome determination. For this question, the tool was adapted for descriptive cohorts (without a comparator group), and the items (S2, S3, and C) were not considered in the evaluation.

The study by Shah et al. (2023) was the only one that received three evaluations, as it addressed the three research questions of this review [21]. The study was assessed using the QUIPS Tool for potential prognostic factors in patients with post-COVID cardiac conditions (Table 4) [21].

Although the risk assessment was balanced, three important domains were classified as high risk. These items reflect the quality of the prognostic factors determination, indicating a possible presence of bias in the results of the statistical analyses. Despite this, the authors characterised the loss observed and concluded the proposed analyses.

## 4. Discussion

Given the known potential of the SARS-CoV-2 virus to cause cardiac damage in the short, medium, and long term, this review aimed to answer the research questions regarding the factors associated with the development of post-COVID cardiac conditions, the incidence of key outcomes, and potential prognostic factors. Nearly 10,000 publications were found, of which seven met the eligibility criteria. All the included publications were original articles and cohort studies, published in English.

Vaccination stood out among the associated factors, with its protective effect, by significantly reducing the risk of developing post-COVID heart disease [17,20]. This effect was particularly favourable for individuals aged 65 and older, even after just the first dose, with a reduction in the occurrence of coronary artery disease [16]. Conversely, COVID-19 reinfections increased the risk of cardiovascular outcomes, indicating the importance of adopting prevention strategies against new infections [18].

The effectiveness of SARS-CoV-2 vaccines in preventing hospitalisation, severe cases, and fatalities is well known [24,25]. Regarding the protection provided by vaccines against post-COVID-19 conditions, specifically in preventing developed cardiac complications, a study conducted in Argentina linked complete vaccination against COVID-19 to a lower incidence of persistent cardiac symptoms in this phase (adjusted OR: 0.52; 95% CI: 0.40–0.69) [26].

Similar results were found in the United States by a study involving HIV patients, in which vaccinated individuals had a lower incidence of post-COVID cardiac conditions compared to unvaccinated individuals (OR: 0.58; 95% CI: 0.4–0.85) [27].

In terms of the primary outcomes for these patients, most recovered; although, some conditions persisted for more than 200 days of follow-up [19,21]. Shah et al. (2023) followed 144 children affected by MIS-C, of whom 85 (59%) exhibited cardiac abnormalities, which persisted in 4 (2.3%) at the end of eight weeks: 3 with coronary artery dilation and 1 with pericardial effusion [21].

Despite the small number of patients in the recovery and non-recovery groups, differences were observed in factors such as age and symptoms during the acute phase. The four children who did not recover were between two and five years old and presented with gastrointestinal symptoms [21]. Nevertheless, the validity of these results is extremely limited due to the elevated risk of bias that was identified in the study.

In a study conducted in Pakistan, Aziz et al. (2023) followed 47 children with MIS-C and identified persistent coronary artery dilation in two patients (20%) at the end of the follow-up period, one of whom had been monitored for seven months [28]. Other studies reported persistent cardiac abnormalities even after several months and suggested continued long-term monitoring of patients to evaluate sequelae [29,30].

Zaoui et al. (2022) followed patients with non-fulminant myocarditis due to COVID-19 who were undergoing treatment for heart failure [30]. Prognostic factors for poor recovery within three months included age over 60 years, troponin levels exceeding 1200 times the normal range, and the occurrence of pericardial effusion [30].

Post-COVID cardiac conditions place a significant burden on healthcare systems due to the essential resources required for patient treatment and intensive care support [31]. Understanding the factors associated with their occurrence and prognosis allows for better resource allocation, planning, and prioritising preventive measures such as vaccination and overall health care.

Since the present study exclusively analysed studies that assessed patients with no previous history of heart disease, it is recommended that future literature reviews investigate the worsening or development of new cardiac conditions in individuals with pre-existing heart diseases. Such studies would provide a more comprehensive understanding of the burden imposed by cardiac conditions related to COVID-19.

This review presents some limitations that should be acknowledged. Among them is the scarcity of published studies that comprehensively address the topic of prognosis, both in its clinical and methodological aspects. Although much has been published about COVID-19 and its implications, few studies met the strict selection criteria, and only seven were included to answer the three research questions.

Most publications were accessible in one of the four languages included in this review. Only two articles were published in languages other than those established by the inclusion criteria, but they could potentially contribute additional information. Similarly, studies conducted without laboratory confirmation of the COVID-19 diagnosis were also excluded. However, the authors of this review aimed to adhere to strict selection criteria, as these specified a well-defined population regarding the presence of COVID-19 and the absence of pre-existing heart diseases that could bias the study results.

The publications included in this review were highly heterogeneous concerning the population analysed, factors studied, and outcomes, therefore precluding the possibility of conducting a meta-analysis. Nevertheless, each study provided valuable insights that enhance the understanding of the complexity of cardiac complications potentially attributed to COVID-19 and highlight measures already underway that may help address these issues.

The strengths of this review include comprehensive searches in accordance with PRISMA guidelines, the execution of all stages in pairs and independently, as well as the use of quality assessment tools such as the modified NOS and the QUIPS Tool for the methodological evaluation of the included studies.

## 5. Conclusions

The present findings provide valuable contributions to a better understanding of the evolution of post-COVID cardiac conditions. Despite the limited number of eligible studies, this review offers insights that describe the progression of cardiac conditions, from their onset to medium-term follow-up of patients.

The protection offered by the COVID-19 vaccination regimen was observed beyond the acute phase of the disease, reducing the risk of developing post-COVID cardiac conditions. Public policies encouraging vaccination should be promoted to prevent SARS-CoV-2 infections and reinfections.

Given that both COVID-19 and heart diseases occupy a significant place on the global health agenda, post-COVID cardiac conditions deserve due attention. Although most patients recover in the short term, some require care for many months to prevent chronicity and complications, particularly in vulnerable groups such as children and older adults.

COVID-19 emerged as a pandemic in 2020, and four years later, it continues to impact the entire planet. This study provides important evidence to guide government policies on post-COVID conditions surveillance, prevention, and targeted healthcare interventions.

Although this review compiles the available evidence on the topic, it is clear that there is still much to learn about post-COVID cardiac conditions. Strengthening the research agenda by proposing and conducting primary studies on the subject is important. Additionally, this review should be regularly updated as new studies are published in the field.

## Figures and Tables

**Table 1:** Main characteristics and conclusions of studies on factors associated with post-COVID cardiac conditions (question 1).

Author (Year) Country	N^o^ of Measurements of the Outcome and Follow-Up Time	Study Population and Subgroups	Characterisation of the Population/Groups	Association Factors and Measures Under Analysis	Cardiac Outcomes Analysed/Results	Results/Conclusions
Bowe et al. (2022) USA [18]	7 observations: T0: positive test T1: 90 days After T1: 30 to 60 days 60 to 90 days 90 to 120 days 120 to 150 days 150 to 180 days	Population: Soldiers using the US VHA (Veterans Health Administration of United States) with at least one positive SARS-CoV-2 test between 1 March 2020 and 6 April 2022 who were alive 90 days after the first test. Exposed: 40,947 soldiers who experienced SARS-CoV-2 reinfection. Unexposed: 443,588 soldiers who were not reinfected with SARS-CoV-2.	Without reinfection: White race/colour (69.54%), male sex (87.22%), never smoked (45.55%), BMI $\bar{x}$ (±): 31.04 kg/m^2^ (±6.05), age $\bar{x}$ (±): 59.84 (±15.74). No vaccination (64.14%), 1 dose (7.99%), 2 doses (20.01%), 3 or more doses (7.86%). Type 2 diabetes (31.98%), hospitalisation during the acute phase of the 1st infection (9.54%). With reinfection: White race/colour (69.12%), male sex (86.4%), never smoked (46.5%), BMI $\bar{x}$ (±): 30.97 kg/m^2^ (±6.1), age $\bar{x}$ (±): 59.97 (±15.76). No vaccination (62.78%), 1 dose (7.52%), 2 doses (22.88%), 3 or more doses (6.82%). Type 2 diabetes (32.52%), hospitalisation during the acute phase of the 1st infection (11.83%).	Vaccination status and reinfection Measures: *p*-value, relative risk (95% CI), and excess risk per 1000 people in 30 days (AR) (95% CI).	Cardiovascular diseases: Dysrhythmias (tachycardia, bradycardia, ventricular arrhythmias, atrial fibrillation, and atrial flutter) Inflammatory heart disease (pericarditis and myocarditis) Ischemic heart disease (acute coronary disease, angina, myocardial infarction, ischemic cardiomyopathy) Other cardiac disorders (heart failure, non-ischemic cardiomyopathy, cardiac arrest, and cardiogenic shock)	Reinfection (regardless of vaccination status) increases the risk of post-COVID cardiovascular conditions: Reinfection x no reinfection 30 to 60 days reinfection AR: 15.37 (95% CI: 12.78; 18.21) RR: 2.19 (95% CI: 1.99, 2.41) 90 to 120 days after reinfection AR: 8.93 (6.91; 11.15) RR: 1.66 (1.51, 1.82)
Wan et al. (2023) Hong Kong [20]	2 observations: acute phase: <28 days and post-acute phase: >28 days	Population: Patients aged ≥18 years with COVID-19 infection between 23 February 2021 and 31 October 2022, according to electronic health records, vaccination records, and data sets of COVID-19 infection records from the Hong Kong Special Administrative Region Groups after weighting ; Exposed: 902,601 Vaccinated for COVID-19; Unexposed: 999,740 unvaccinated	No vaccine Males: 438,139 (43.8%); Diabetes: 95,750 (9.6%); Severe COVID-19: 1209 (0.1%); Age $\bar{x}$ (s±): 49.2 (±20.2) 1 dose Males: 439,332 (44.0%); Diabetes: 97,334 (9.7%); Severe COVID-19: 1196 (0.1%); Age $\bar{x}$ (s±): 49.5 (±19.2) 2 doses Males: 429,225 (43.4%); Diabetes: 97,677 (9.9%); Severe COVID-19: 1222 (0.1%); Age $\bar{x}$ (s±): 50.6 (±17.3) 3 doses Males: 430,408 (43.5%); Diabetes: 99,217 (10.0%); Severe COVID-19: 1426 (0.1%); Age $\bar{x}$ (s±): 50.6 (±16.1)	Vaccine doses, age, sex, Charlson comorbidity index (CCI), disease severity, and type of vaccine; Measures: Incidence rate, relative risk, and their respective (95% CI)	Coronary heart disease RR (95% CI) ≥ 65 years 1 dose: 0.75 (0.60; 0.94) More than 90 days 2 doses: 0.73 (0.61; 0.88) Heart failure More than 90 days 1 dose: 0.67 (0.53; 0.85) CCI < 4 2 doses: 0.41 (0.29; 0.57) CCI ≥ 4 2 doses: 0.74 (0.59; 0.91)	Coronary artery disease: 1 vaccine dose reduces the risk in individuals aged 65 years or older, while 2 vaccine doses reduce the risk of occurrence regardless of age, sex, CCI, or vaccine type, even after 90 days post-infection. Heart failure: 1 vaccine dose reduces the risk of occurrence regardless of age, sex, CCI, or vaccine type, even after 90 days post-infection. On the other hand, 2 vaccine doses confer greater protection to individuals with CCI < 4 compared to those with CCI ≥ 4
Shah et al. (2023) India [21]	7 observations: 1, 2, 4, 7, and 8 weeks and 3 and 6 months (number and times varied between study participants with only two children followed at 6 months)	Population: 144 sequential patients diagnosed with MIS-C treated by the authors at cardiology Exposed: 85 patients with cardiac abnormalities Unexposed: 59 patients without cardiac abnormalities	Population (144 children with MIS-C) Males: n = 93 (64.6%); Weight: $\bar{x}$: (s±) = 20.5 kg (±14.7); Symptom: fever n = 137 (95.1%); Intensive Care: n = 76 (52.8%). Age groups (%): <12 months: 17 (11.8%); 12–60 months: 60 (41.7%); 60–120 months: 44 (30.6%); >120 months: 23 (16.0%) Children with cardiac abnormalities (85) Males: n = 56 (65.9%); Weight: $\bar{x}$ (s±)’ 22.5 (±14.3); Intensive Care: n = 49 (57.6%) Age groups (%): <12 months: 6 (7.1%); 12–60 months: 39 (45.9%); 60–120 months: 25 (29.4%); >120 months: 15 (17.6%)	Sex, age, symptom presentations, laboratory results, duration between COVID-19 infection and current disease, echocardiographic findings, electrocardiographic findings, and management. Measures: Incidence and *p*-value	Ventricular dysfunction, coronary dilatation, pericarditis, atrioventricular valve regurgitation, and conduction abnormalities	No association was found between the factors analysed and the outcomes studied ^a^ (NT Pro-BNP elevated showed cardiac abnormality *p*-value = 0.09)
Zisis et al. (2022) USA [17]	3 observations: baseline (diagnosis) 28 days 3 months	Population: Adult patients aged ≥ 18 years with SARS-CoV-2 infection (confirmed by PCR) who sought care in the United States with medical records on the TriNetX Research Network platform and # of patients diagnosed with COVID-19 at least one week after administration of the full vaccine were included in the vaccinated cohort. Exposed: 25,225 vaccinated for COVID Unexposed: 25,225 unvaccinated	Vaccinated: Females n: 15,094 (59.84%) ; White race/colour n: 17,266 (68.45%) ; Hypertension n: 11,974 (47.36%); Diabetes n: 5774 (22.89%); BMI $\bar{x}$ (s±): 30.20 kg/m^2^ (±7.33); Unvaccinated: Females n:15,129 (59.98%); White race/colour n: 17,381 (68.90%); Hypertension n: 11,963 (47.43%); Diabetes n: 5698 (22.59%); BMI $\bar{x}$ (s±): 30.68 kg/m^2^ (±7.40)	Vaccination Measures: incidence, relative risk (RR), and attributable risk (AR) in 28 days and 90 days	Grouped heart diseases: ICD-10 (I30–52, I21)	Full vaccination protects against the occurrence of post-COVID cardiac conditions at 28 and 90 days after infection: 28 days: RR (95% CI): 0.49 (0.43, 0.57) AR (95% CI): −15.76 (−18.96; −12.57) 90 days: RR (95% CI): 0.35 (0.29; 0.44) AR (95% CI): −13.07 (−15.55; −10.60)

Note: T0 (date of first positive SARS-CoV-2 test); T1 (date of reinfection); BMI (body mass index); AR (attributable risk or risk difference); RR (relative risk); CCI (Charlson comorbidity index); ^a^ Shah et al. (2023) [21] did not present the table of statistical results; MIS-C (multisystem inflammatory syndrome in children associated with COIVD-19); PCR (polymerase chain reaction); ICD-10 (International Classification of Diseases—10th Revision); NT Pro-BNP (blood marker indicating the degree of heart failure).

**Table 2:** Main characteristics and conclusions of studies on the prognosis of patients with post-COVID cardiac conditions (question 2).

Author Year Country	N^o^ of Outcome Measurements in the Post-COVID Period	Study Population and Subgroups	Validation Criterion of Cardiac Condition Post-COVID	Cardiac Outcomes Analysed (Measures)	Main Outcomes	Conclusions
Clark et al. (2021) USA [19]	Group 1 (patients with cardiopulmonary symptoms in the late convalescence phase) 3 measurements (in days): 1st ranged from 33 to 124 2nd ranged from 82 to 271 3rd ranged from 119 to 245	Soldiers in active service or recent military retirement (previous year), who routinely exceed 6 h of strenuous activity per week Group 1: 50 soldiers who had COVID-19 and remained with cardiopulmonary symptoms in the late convalescence phase of recovery	Physical examination, ECG, and CMR	Myocarditis Pericarditis Takotsubo cardiomyopathy Biventricular systolic dysfunction (incidence and time to resolution of cardiac sequelae)	Patients with post-COVID cardiac condition: Myocarditis—4 cases (8%): 1 acute myocarditis, 3 with healing myocarditis, 1 of which (2%) had concomitant pericarditis; Takotsubo cardiomyopathy—1 case (2%); new biventricular systolic dysfunction without myocarditis—1 case (2%) Follow-up of myocarditis patients: Case 1: LGE1: 8.8% (33 d from COVID diagnosis): LGE2: 2.8% (82 d); LGE3: 0 (119d); Case 2: LGE1: 3.5% (124 d); LGE2: 3.5% (271 d); Case 3: LGE1: 5.1% (38 d); LGE2: 5.1% (122 d); LGE3: 5.1% (213 d); Case 4: LGE1: 7.4% (97 d); LGE2: 2.1% (192 d); LGE3: 0 (245 d).	Follow-up CMR among cases of myocarditis showed two patterns: recovery with gradual resolution of LGE (2 cases) and persistence (2 cases), with one soldier presenting with continuous active myocarditis with T2 elevation more than 7 months after COVID-19 diagnosis.
Shechter, A et al. (2022) Israel [22]	2 measurements 60 days from negative PCR Median = 142 (IQR: 111; 197) days after COVID-19 diagnosis.	87 patients (≥18 years) who had PCR+ for COVID-19, with persistent symptoms after 60 days of negative PCR (formal recovery), have a written referral from their attending physicians stating the exact manifestation(s) believed to be CV disease, attended between June 2020 and June 2021. Post-COVID cardiac conditions: 9 patients (10 events)	Cardiac provocation test, Holter ECG, cardiopulmonary exercise test (CPET), CCT, CMR, PFT, and chest HRCT	New CV diagnoses potentially related to COVID-19, major adverse cardiovascular events (MACE), defined as acute coronary syndrome, acute stroke, and CV death. (Incidence = absolute number per follow-up visit.)	Initial diagnoses: 10 cases; Myocarditis: 3 cases; Myopericarditis: 2 cases; Chronotropic incompetence: 3 cases; Unperturbed sinus tachycardia: 1 case; Atrial tachycardia: 1 case. At 1-year follow-up (142 days = median): Complete resolution of cases = Recovery of 100% of cases at a median of 3 months.	Recovery of 100% of cases at a median of 3 months. No MACE or death recorded.
Shah et al. (2023) India [21]	7 observations: 1 week 2 weeks 4 weeks 7 weeks 8 weeks 3 months 6 months (number and times varied between study participants with only two children followed at 6 months)	Population: 144 sequential patients diagnosed with MIS-C seen by the authors at cardiology Patients with cardiac abnormalities: 85	Echocardiogram: confirmation of one of the five abnormalities at any of the visits	Ventricular dysfunction, coronary dilatation, pericarditis, atrioventricular valve regurgitation, and conduction abnormalities. Other outcomes: coronary aneurysm. Incidence = absolute number per follow-up visit; average duration of recovery (in days).	Abnormal electrocardiogram: 39 patients; 1 week: 39; 2 weeks: 3; 4 weeks: 3; 7 and 8 weeks: 0 Pericardial effusion: 25 patients; 1 week: 22; 2 weeks: 6; 4 weeks: 1; 7 and 8 weeks: 1 Coronary artery dilatation: 37 patients; 1 week: 31; 2 weeks: 24; 4 weeks: 11; 7 weeks: 4; 8 weeks: 3 left ventricular dysfunction: 20 patients; 1 week: 20; 2 weeks: 3; 4 weeks: 1; 7 and 8 weeks:0 Atrioventricular artery regurgitation: 33 patients; 1 week: 33; 2 weeks: 2; 4 weeks: 3; 7 weeks: 2; 8 weeks: 0	51 children completed the follow-up (Loss = 40%). Recovery: 92% (47/51) of children; median of 30 (range: 22 to 37 days) (Kaplan–Meier analysis). Ventricular dysfunction: resolved and no child presented cardiac dysfunction at the time of hospital discharge. Pericardial effusion: completely resolved, except one, before discharge. Atrioventricular valve regurgitation correlated with ventricular dysfunction and disappeared completely when LV function improved. Three children: coronary artery dilatation persisting at the end of 8 weeks: 1 moderate left coronary artery aneurysm persisting at 182 days; 2 children: small left coronary artery aneurysm, persisting after 168 and 201 days each.
Cassar et al. (2021) UK [23]	2 measurements after infection: 2/3 months; 6 months	58 patients with confirmed moderate-to-severe COVID-19 who were admitted for at least 48 h to the Oxford University Hospitals National Health Service Foundation Trust (14 March and 25 May 2020)	ECG for each participant and interpreted according to the Minnesota Code Manual of Electrocardiographic Findings; Cardiopulmonary MRI	Coronary artery disease; atrial fibrillation, myocardial infarction, myocarditis, and pericardial effusion. (CVD incidence at 2/3 months and 6 months)	Results at 2/3 months and at 6 months: coronary artery donation: 2/58 and 1/46; atrial fibrillation: 1/58 and 1/46 (same patient); myocarditis pattern: 6/52 (11.5%) and 5/43 (11.6%); myocardial infarction: 1/52 (1.9%) and 0/43 (0.0%); pericardial effusion: 1/52 (1.9%) and 0/43 (0.0%).	Partial resolution of events. None of the patients met Lake Louise’s updated criteria for active myocarditis.

Note: ECG (electrocardiogram); CMR (cardiac magnetic resonance); LGE (late gadolinium enhancement); PCR (polymerase chain reaction); CV (Cardiovascular); CPET (cardiopulmonary exercise test); CCT (cardiac computed tomography); PFT (pulmonary function test); HRCT (high-resolution computed tomography); MACE (major adverse cardiovascular events); MIS-C (multisystem inflammatory syndrome in children associated with COIVD-19); LV (left ventricular); CVD (cardiovascular disease).

**Table 3:** Critical evaluation of studies—Newcastle–Ottawa Scale (NOS) for cohort assessment.

Studies—Question 1	Items								
	Selection				Comparability	Outcome			Score
	S1	S2	S3	S4	C	D1	D2	D3	
Shah et al. (2023) [21]	0	*	*	*	0	*	*	*	6 of 9
Wan et al. (2023) [20]	*	*	*	*	**	*	*	*	9 of 9
Zisis et al. (2022) [17]	*	*	*	0	**	*	*	*	8 of 9
Bowe et al. (2022) [18]	0	*	*	*	**	*	*	*	8 of 9
**Studies—Question 2**	**S1**	**—**	**—**	**S4**	**—**	**D1**	**D2**	**D3**	**Score**
Clark et al. (2021) [19]	0	—	—	0	—	*	*	*	3 of 5
Shah et al. (2023) [21]	0	—	—	*	—	*	*	*	4 of 5
Shechter et al. (2022) [22]	0	—	—	*	—	*	*	*	4 of 5
Cassar et al. (2021) [23]	0	—	—	0	—	*	*	*	3 of 5

Note: NOS Scale: * Star for each item met; S1 representativeness of the exposed cohort; S2 selection of the non-exposed cohort; S3 verification of exposure; S4 demonstration that the outcome of interest was not present at the start of the study; C controls for confounding factors; D1 outcome assessment; D2 follow-up was long enough for outcomes to occur; D3 adequacy of follow-up. Each (*) indicates good quality in the item evaluated, and (0) represents no score (—). For the studies in question 2, items 2 and 3 of the selection and the comparability item were not considered in the final score, as they do not apply to the research question (descriptive only).

**Table 4:** Critical evaluation of the study by Shah et al. (2023) [21]—QUIPS Tool (quality of prognostic studies).

Item	Assessment	Observations
1 Participation	Moderate risk	Lack of clarity on the representativeness of the population
2 Attrition	High risk	Loss of 40% described with important differences in PFs and outcomes measured between adherent and non-adherent groups
3 PF measurement	Low risk	Data collected from medical records and standardised measurements
4 Outcome measurement	Low risk	
5 Confusion	High risk	Method for controlling bias in the study not informed
6 Statistical analysis	High risk	High probability of error due to lack of representativeness of the compared groups (PF analysis).
Final analysis	High risk	High risk (3 important items in the PF analysis were classified as high risk)

Note: PF = prognostic factor. Item 1: multicentre study, but without definition of recruitment centres and criteria (inclusion and exclusion). We are not sure of the representativeness of the study population. Perspective of 1 paediatric cardiologist. Item 2: there was a loss of 40%. Although the loss was compared and justified, many outcomes were considered undefined. In addition, important characteristics (respiratory symptoms and coronary artery dilation) were different between adherents and non-adherents. Item 3: PF data were collected from patients’ medical records and standardised measurements. Item 4: data collected from medical records and standardised measurements. Item 5: method for bias control in the study is not informed, nor is the measure adjusted. Item 6: in the study of prognostic factors, statistical analysis compares a very small group with another group. We cannot affirm that it is adequate since the n is small. The probability of error is high, due to lack of representativeness and statistical capacity. Final analysis—important items for assessing study quality may be compromised.

## Data Availability

The data supporting the conclusions of this study are available in the Supplementary Materials.

## Supplementary Materials

The following supporting information can be downloaded at: https://www.mdpi.com/article/10.3390/life15030388/s1, Table S1: Description of the PECOS strategy for the formulation of guiding questions for the systematic review; Table S2: Search strategy conducted in the BVS, EBSCOhost, Embase, MEDLINE databases and grey literature, on 16 December 2023; Table S3: Complete extraction spreadsheet for question 1; Table S4: Complete extraction spreadsheet for question 2; Check list PRISMA_Maria et al.
